# Predictive modelling of mandibular osteoradionecrosis in head and neck cancer patients: clinical and dosimetric insights

**DOI:** 10.1007/s00784-025-06385-3

**Published:** 2025-05-26

**Authors:** Julian Leeder, Ali Modabber, Frank Hölzle, Michael J. Eble, Ahmed Allam Mohamed

**Affiliations:** 1 Department of Radiation Oncology, Medical Faculty, RWTH Aachen University, Pauwelstraße 30, Aachen, 52074 Germany; 2 Department of Oral and Maxillofacial Surgery, Medical Faculty, RWTH Aachen University, Aachen, Germany; 3 Center for Integrated Oncology Aachen, Bonn, Cologne, and Duesseldorf (CIO ABCD), Aachen, Germany

**Keywords:** Intensity-modulated radiotherapy, Radiotherapy toxicity, Post-radiotherapy dental extraction, Nomogram development

## Abstract

**Introduction:**

Mandibular osteoradionecrosis (ORN) is a serious complication of radiotherapy (RT) for head and neck cancer (HNC), with an incidence of 3–15%. ORN results from radiation-induced bone necrosis and may require surgical intervention. This study investigates clinical and dosimetric predictors of ORN risk and develops a predictive model for individualized risk assessment.

**Materials and methods:**

This retrospective case-control study included 298 HNC patients treated with RT or chemoradiotherapy between January 2012 and May 2020. Dosimetric parameters, including mandibular V10–V60, mean dose (Dmean), and maximum dose (Dmax), were analyzed alongside clinical data such as age, tumor site, smoking history, and dental extractions.

**Results:**

Over a median follow-up of 32.4 months, 20 patients (6.7%) developed ORN, with a 5-year cumulative incidence of 7.4%. Multivariate analysis identified mandibular V50 (HR = 1.05, *p* = 0.0015) and post-RT dental extractions (HR = 2.51, *p* < 0.0001) as significant ORN risk factors, while age was protective (HR = 0.96, *p* = 0.047). A V50 cutoff of 25.4 cm³ was most predictive (*p* = 0.0016). The multivariate model incorporating V50, age, and dental extractions demonstrated strong accuracy (C-index: 0.815, AUC: 0.8).

**Conclusion:**

V50 and post-RT dental extractions are key predictors of ORN. The developed nomogram enables personalized risk assessment, supporting treatment optimization. These findings emphasize the need for tailored RT planning and dental care to mitigate ORN risk, warranting validation in multi-institutional cohorts.

**Supplementary Information:**

The online version contains supplementary material available at 10.1007/s00784-025-06385-3.

## Introduction

Mandibular osteoradionecrosis (ORN) is one of the earnest complications arising after radiotherapy (RT) in patients treated for head and neck cancers (HNC) [1, 2]. It is characterized by clinical or radiological evidence of bone necrosis within the radiation field that persists without evidence of healing at least three to six months after treatment, and it is not associated with tumor recurrence [3].

The reported incidence of mandibular ORN varies, but it generally ranges between 3% and 15% in patients undergoing RT for HNC [3–5]. The underlying pathology of ORN is multifactorial and primarily related to radiation-induced damage to bone and soft tissue structures. Radiation disrupts the normal homeostasis of bone remodeling, leading to vascular damage, hypoxia, fibrosis, and subsequent bone necrosis [6, 7]. The mandible is highly susceptible to ORN due to its limited blood supply compared to other bones in the craniofacial region, making it more vulnerable to radiation-induced ischemia [8]. Once the bone becomes devitalized, secondary infection and progressive necrosis risk increase, further complicating healing [7, 9].

The exact etiology remains unclear. However, in the literature, several factors have influenced the incidence of ORN, including radiation dose to the mandible and treatment modalities [10–12] with some studies reporting that higher radiation doses to the mandible significantly increase the risk of ORN when doses exceed 50 Gy [13, 14]. Also, dental extractions and periodontitis can dramatically increase the likelihood of ORN [15]. However, the optimal timing of dental extractions around the course of RT remains controversial [16], with no clear consensus on whether extractions should be performed exclusively before RT or can be safely done after RT [17]. On one hand, removing infectious foci before RT is generally recommended to prevent later complications; on the other hand, extraction wounds may themselves succumb to compromised healing post-irradiation, contributing to ORN risk [17].

Despite current knowledge, there is no consensus or clear recommendations to prevent ORN, and its occurrence remains a significant concern, necessitating ongoing research to better understand its pathophysiology and identify more effective prevention and management strategies [18].

In this retrospective analysis, we aimed to investigate possible clinical and dosimetric factors associated with increased risk for mandibular ORN and establish a nomogram to facilitate a predictive tool.

## Materials and methods

### Study design and patient inclusion

This retrospective study included patients diagnosed with HNC treated at the Department of Radiation Oncology, University Hospital Aachen, between January 2012 and May 2020. All patients underwent RT or radiochemotherapy (RCT) with curative intent for primary tumor site and cervical lymphatics using IMRT as step and shoot 2012–2013 and Volumetric Modulated Arc Therapy (VMAT) 2013–2020.

The inclusion criteria were: (1) a confirmed diagnosis of carcinoma in the Nasopharynx, oral cavity/oropharynx, larynx/Hypopharynx, or salivary gland, or carcinoma of unknown origin (CUP) (2). Adjuvant or definitive RT should be received with a minimum prescribed dose of 50 Gy for the primary and lymphatics (3). a follow-up period of at least 6 months post-RT.

All patients with HNC at our academic center, part of a certified cancer center network (CIO ABCD), scheduled for radiotherapy underwent a standardized pre-treatment dental screening program, including dental radiographs. This consisted of a comprehensive dental assessment to identify and eliminate potential oral infection foci, with any necessary dental sanitation procedures or extractions of compromised teeth performed before the start of RT. After dental extractions, a healing period of approximately two weeks was allowed to ensure adequate wound closure before initiating RT.

Dentulous Patients and patients with implants were offered fluoride treatments during RT, and oral hygiene during and after RT was instructed to the patients.

Following RT, Tumor follow-up is scheduled every 3 months for the first two years and then every six months for an additional 3 years, followed by annual checks. Dental follow-up was conducted locally at the center or by the general dentists for lifelong routine dental care. If dental extractions became necessary in the years after RT, they were recommended to be performed with antibiotic prophylaxis and local wound coverage, ideally at our center, to minimize the risk of ORN.

**Fig. 1:**
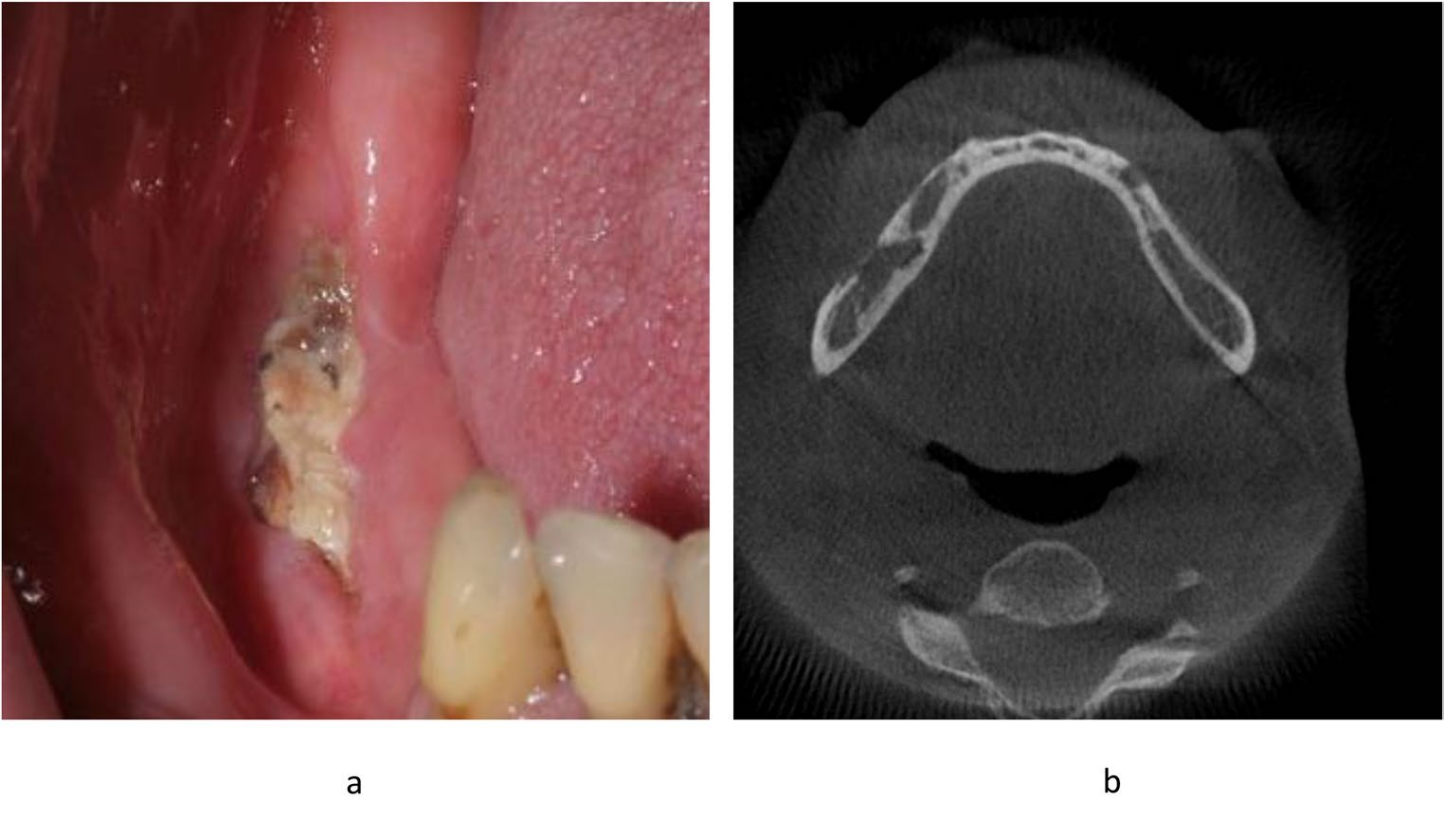
Clinical and radiological presentation of mandibular osteoradionecrosis (ORN). (**a**) an intraoral photograph shows exposed necrotic bone along the lingual surface of the mandible, with surrounding mucosal inflammation, (**b**) Axial CT scan revealing areas of cortical bone destruction and sequestration in the mandible, consistent with ORN

During the follow-up, mandibular ORN was identified as an exposed bone within a previously irradiated area that had not undergone healing over three months in the absence of any signs of persistent or recurrent tumor [3]. Clinically, diagnosis required either persistent bone exposure or chronic mucosal ulceration overlying necrotic bone. Radiologically, ORN was defined by signs of osseous necrosis, including irregular osteolytic bone destruction, cortical disruption, bone sequestration, or pathological fracture [2, 3, 19]. The severity of ORN was staged according to the Notani classification system [20]. In this system, Grade I is defined as ORN confined to the alveolar bone; Grade II includes ORN involving the alveolar bone and/or the mandible above the level of the mandibular canal; and Grade III is characterized by extension of ORN below the mandibular canal level, often associated with skin fistula formation and/or the presence of a pathological fracture (Fig. 1).

Clinical data were retrieved from the internal database to determine whether patients developed mandibular ORN during routine follow-ups in the Departments of Radiation Oncology and Oral and Maxillofacial Surgery.

### Dosimetric and volumetric analysis

Original radiotherapy plans were restored from the Pinnacle3 treatment planning system (V.14.0; Philips Healthcare, Amsterdam, Netherlands) for detailed dosimetric analysis. Each patient’s mandible was contoured using their planning CT scans, and dose-volume histograms (DVHs) were generated. The following dosimetric parameters were extracted: total mandibular volume (cm³), prescribed dose (PD), mean dose to the mandibula (Dmean), dose near maximum to the mandibula (D2%), maximum dose to the mandibula (Dmax), and volumetric thresholds ranging from V10Gy to V60Gy (cm³).

### Data collection

Clinical data collected for each patient included age, gender, tumor site, tumor stage, treatment details (RT/RCT), follow-up duration, smoking status, and dental extraction history; dental extractions were defined in this study as tooth removal performed either electively before Radiotherapy (pre-RT) or non-electively after radiotherapy (post-RT), including those due to dental complications such as caries or infection. Spontaneous tooth losses due to trauma or periodontitis without dental intervention were not recorded. These clinical variables were cross-referenced with the dosimetric parameters to assess potential risk factors for ORN development.

The local ethics committee approved the analysis (Faculty of Medicine, RWTH Aachen University, EK 24–383).

### Statistical analysis

The primary endpoint of the study was the occurrence of mandibular ORN following radiotherapy. Secondary endpoints included identifying dosimetric and clinical risk factors for ORN development. The association between dosimetric parameters and ORN was examined using dose-volume histograms and volumetric measures.

Descriptive statistics were presented as medians for continuous variables and frequencies and percentages for categorical variables. The Mann-Whitney U test compared non-parametric data between the ORN and non-ORN groups.

Receiver operating characteristic (ROC) curve analysis was used to identify optimal cut-points for predicting ORN risk. Kaplan-Meier survival analysis was performed to estimate the cumulative incidence of ORN, with group differences assessed by the log-rank test. Univariate and multivariate Cox proportional hazards models were used to calculate hazard ratios (HR) and 95% confidence intervals (CI) for clinical and dosimetric predictors of ORN.

Concordance (c) statistics were calculated to assess the predictive accuracy of the models. Time-dependent ROC curve analysis and area under the curve (AUC) calculations were performed to evaluate the predictive accuracy of different models using the “timeROC” package. Nomograms were developed to predict the 5-year risk of ORN, and calibration curves were used to evaluate the model’s calibration. The “rms” package was used for nomogram development, with 1000 bootstrap resamples applied for internal validation.

All statistical analyses were conducted using R software version 4.3.1. A two-sided p-value of < 0.05 was considered statistically significant. The study reporting followed STROBE guidelines for observational studies, and model development followed TRIPOD recommendations for prognostic models (details in Appendix 1 & 2).

## Results

### Baseline characteristics

**Fig. 2:**
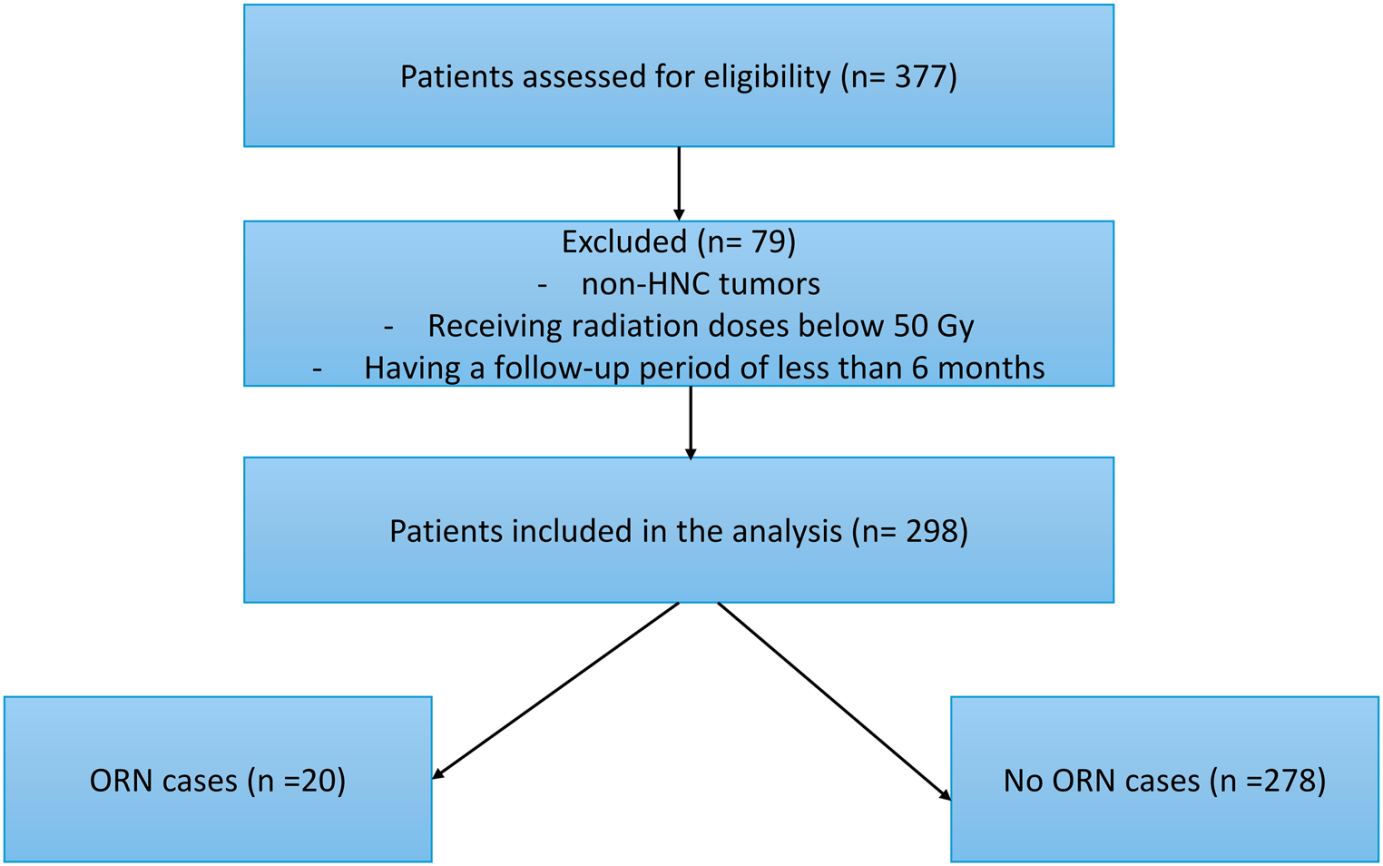
Flowchart of the patients included in the analysis

A total of 377 patients who received radiotherapy for the head and neck region during the aforementioned period were identified. Of these, 79 patients were excluded due to having non-HNC tumors, receiving radiation doses below 50 Gy, or having a follow-up period of less than 6 months. The remaining 298 patients were included in the final analysis (Fig. 2). The median follow-up time for these patients was 32.43 months (IQR: 15.27–55.83 months).

**Fig. 3:**
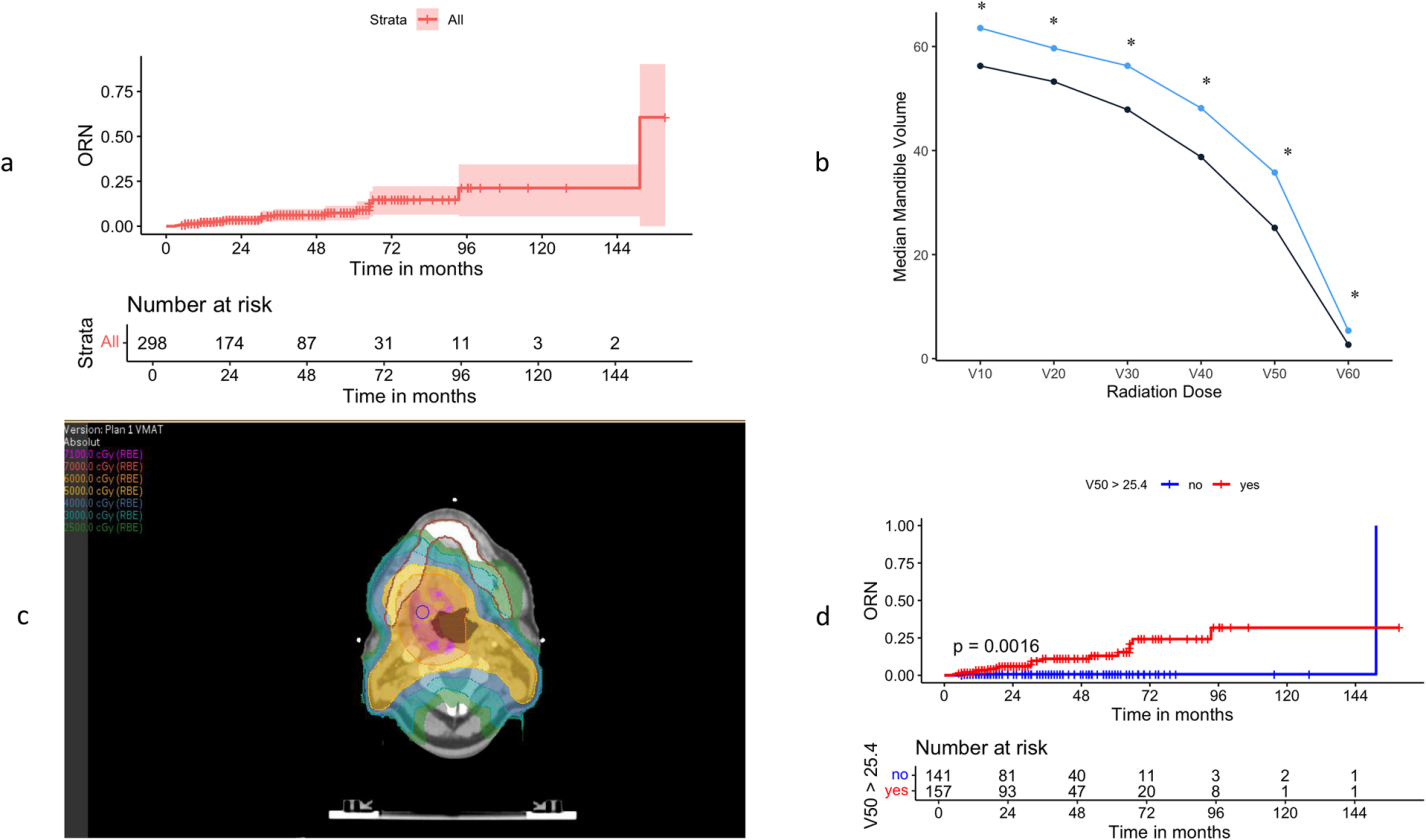
(**a**) The Kaplan-Meier curve illustrates the risk of developing mandibular ORN over time. (**b**) The graph shows the difference in median V10-V60 between patients with and without ORN, *: *p* < 0.05 (Mann-Whitney test). (**c**) Representative axial slice of radiation plan using volumetric modulated arc therapy (VMAT) for a patient with oropharyngeal cancer showing the mandible (brown contour) and color-wash of absolute dose distribution. The yellow isodose line corresponds to 50 Gy. Higher doses (pink, purple) cover the primary tumor. (**d**). The Kaplan-Meier curve demonstrates the difference in ORN risk based on mandibular V50 values (≤ 25.4 cm³ vs. > 25.4 cm³), with a log-rank test result of *p* = 0.0016

Among the 298 patients analyzed, 20 (6.7%) developed mandibular osteoradionecrosis (ORN) (Fig. 2), resulting in a 5-year cumulative incidence of 7.4% (Fig. 3a) (Appendix 2). The median age of all patients was 70 years, and the median mandible volume was 59.55 cm³. The median prescribed radiation dose was 60 Gy, the median mean dose to the mandible (Dmean) was 45.1 Gy, and the median dose to 2% of the mandible (D2%) was 60.6 Gy.

### Dosimetric and volumetric parameters

Differences in baseline characteristics between the two groups are presented in Table 1. The median mandibular volume differed significantly between patients who developed ORN and those who did not, with volumes of 67.1 cm³ and 58.8 cm³, respectively (*p* = 0.0093). The median age was also significantly lower in patients with ORN than those without (66 vs. 71, *p* = 0.0062).

There were no significant differences in the median prescribed radiation dose, Dmean, D2%, or Dmax between patients who developed ORN and those who did not. However, analysis of the absolute volumetric differences in dose-volume parameters (V10 to V60) revealed statistically significant differences between the two groups (Fig. 3b, c).

**Table 1:** Characteristics of patients with and without mandibular osteoradionecrosis (ORN)

Parameter	Patients with ORN (*n* = 20) ( median)	Patients without ORN (*n* = 278) (median)	*P*-Value
Age	66	71	0.00616 ^±^,^*^
Mandibular volume cm³	67.1	58.8	0.0093 ^±, *^
Prescribed dose (Gy)	60	60	0.2865 ^±^
Dmean (Gy)	46.9	44.9	0.071 ^±^
D2% (Gy)	60.7	60.6	0.2795 ^±^
Dmax (Gy)	62.5	62.2	0.534 ^±^
V10 cm³	63.54	56.26	0.0074 ^±,*^
V20 cm³	59.66	53.25	0.0051 ^±^, ^*^
V30 cm³	56.3	47.87	0.0073 ^±^,^*^
V40 cm³	48.15	38.76	0.0028 ^±,*^
V50 cm³	35.77	25.1	0.0034 ^±^,^*^
V60 cm³	5.4	2.7	0.038 ^±^,^*^
Smoking history:			
• Yes	11	125	
• No	9	153	0.38 ^¶^
Dental extractions:	16	83	
• Pre-RT	5	66	
• Post-RT	11	24	0.001 ^¶,*^
Tumor site:			
• Nasopharynx	1 (5%)	17 (6.1%)	
• Oral cavity/Oropharynx	13 (65%)	173 (62.2%)	
• Hypopharynx/Larynx	1 (5%)	22 (7.9%)	
• Salivary Gland	3 (15%)	32 (11.5%)	
• CUP	2 (10%)	34 (12.3)	
Radiation treatment			
• Primary	5	90	0.49 ^¶^
• adjuvant	15	188	

Dmean: mean dose to the mandible; D2%: dose near minimum to the mandible; Dmax: maximum dose to the mandible; V: volume; CUP: carcinoma of unknown primary. ^±^ Mann-Whiteny test: Chi-square test; * indicates p-value < 0.05

### Univariate and multivariate analyses

The univariate analysis examined several factors in relation to ORN development. Age was significantly associated with ORN (HR = 0.96, *p* = 0.021), indicating a protective effect with increasing age. Mandibular volume showed a trend toward significance (HR = 1.03, *p* = 0.0577). The mean dose to the mandible also approached significance (HR = 1.06, *p* = 0.0511), suggesting a potential dose-response relationship (Table 2).

**Table 2:** Univariate and multivariate analysis

	Unvariate: HR (*p*-value)	Multiavaite: HR(*p*-value)
Age	0.96 (0.021)*	0.96 (0.047 *)
Tumor location	0.96 (0.8)	
Mandibular volume	1.03 (0.058)	
Total prescribed dose	1.095 (0.106)	
Mean dose to the mandible	1.06 (0.0511.)	
Dose maximum to the mandible	1.05 (0.331)	
D2% of the Mandible	0.078 (0.199)	
V10 Gy	1.03 (0.0497 *)	
V20 Gy	1.04 (0.0342 *)	
V 30 Gy	1.03 (0.0337 *)	
V 40 Gy	1.04 (0.0131 *)	
V 50 Gy	1.04 (0.00673 *)	1.05 (0.0015 *)
V 60 Gy	1.06 (0.0536.)	
Pre-RT dental extraction	1.66 (0.343)	
Post-RT dental extraction	2.53 (0.000051 *)	2.514 (< 0.000001 *)
Smoking history	1.6 (0.3)	

* indicates p-value < 0.05

Regarding dose-volume parameters, predefined specific thresholds of irradiated volumes were significantly associated with ORN development, V10 Gy (HR = 1.03, *p* = 0.0497), V20 Gy (HR = 1.04, *p* = 0.0342), V30 Gy (HR = 1.03, *p* = 0.0337), V40 Gy (HR = 1.04, *p* = 0.0131) and V50 Gy (HR = 1.04, *p* = 0.0067), were all significantly associated with an increased risk of ORN. The HR for V60 Gy (1.06) was marginally significant (*p* = 0.0536). Additionally, dental extraction after radiotherapy showed a robust association with ORN (HR = 2.515, *p* < 0.0001). Among the 20 patients with ORN (Table 1), 11 experienced post-RT Extraction, of whom 10 (50%) experienced multiple post-RT dental Extractions, and one patient (5%) experienced a single Extraction. This suggests that the extent of dental trauma post-RT, particularly multiple extractions, may be associated with an elevated ORN risk. Further, dental extraction before radiotherapy was not associated with increased hazard for ORN (HR 1.6592, *p* = 0.343) (Table 2).

In the multivariate Cox regression analysis, which adjusted for potential confounding factors, age remained significantly protective (HR = 0.958, *p* = 0.047). The volume of the mandible irradiated at V50 Gy (HR = 1.054, *p* = 0.0015) was significantly associated with increased ORN risk, highlighting this parameter as a critical dose threshold. Dental extraction after radiotherapy showed a robust association with ORN (HR = 2.515, *p* < 0.0001), underscoring the heightened risk associated with post-RT dental procedures (Table 2).

### Determination of optimal cutoff volume for V50 Gy

Given that V50 Gy showed the most significant HR in dose-volumetric analysis, we analyzed the optimal cutoff volume associated with the risk of developing mandibular ORN. The best cutoff volume for V50 Gy was identified as 25.4 cm³, with a sensitivity of 90%, a specificity of 50.4%, and an AUC of 0.696.

Patients with a V50 Gy volume above 25.4 cm³ had a significantly higher cumulative incidence of mandibular ORN than those below this threshold (*p* = 0.0016) (Fig. 3c).

### Cox proportional hazards model and nomogram for ORN prediction

**Fig. 4:**
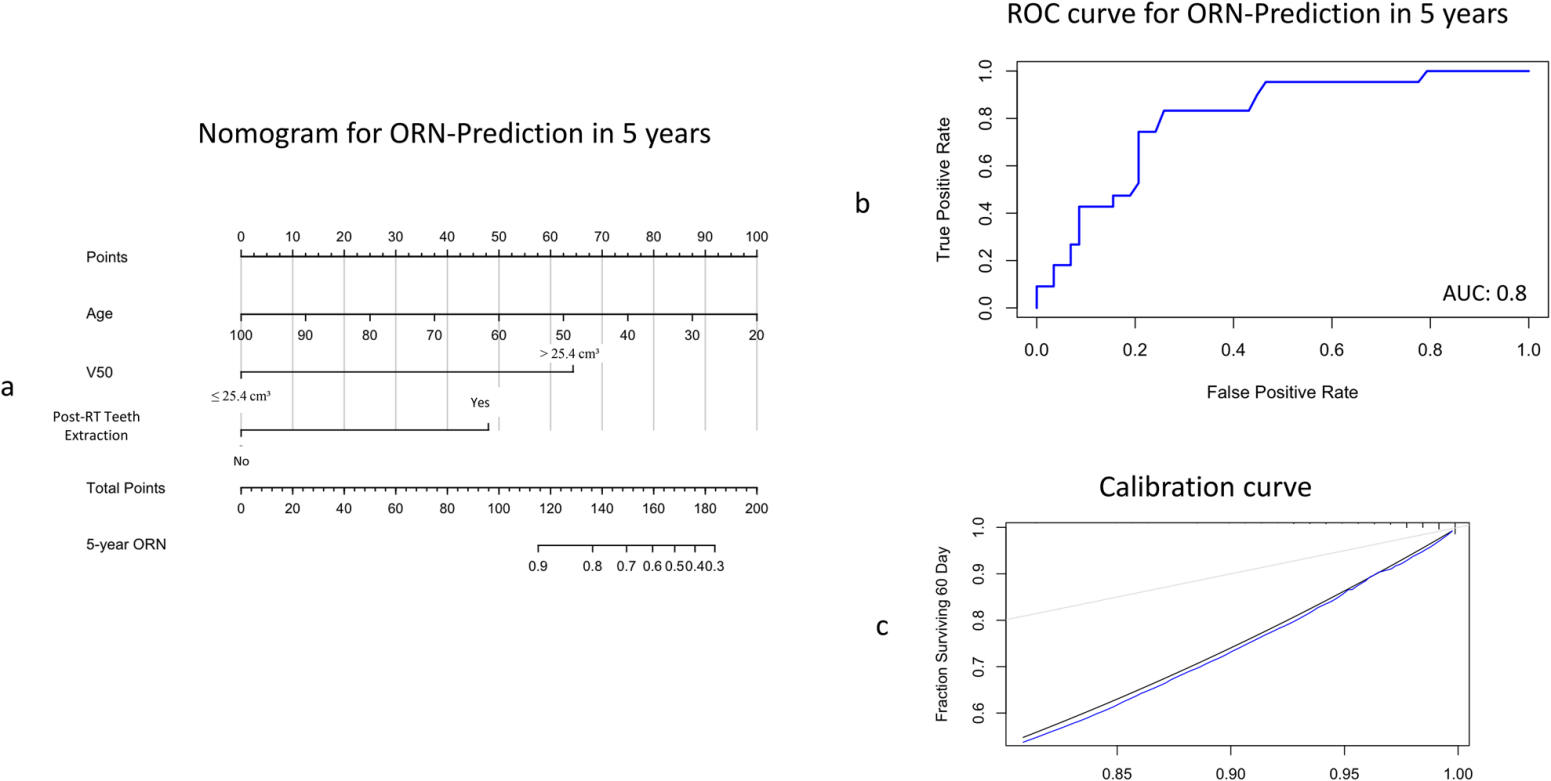
(**A**) Nomogram for predicting the 5-year ORN probability, based on Age, V50 (≤ 25.4 cm³ vs. > 25.4 cm³), and post-RT teeth extraction. For each patient, the total score was the sum of points for these three factors identified on the points scale. Each patient’s 5-year ORN probability was then determined on the total points scale. (**B**) ROC curve respecting the 5-year predictive performance of the model. (**C**) Calibration curve for the nomogram’s observed and predicted 5-year ORN risk using the nomogram, using 1000 bootstrap resamples. The Black Line represents observed ORN risk, the Blue Line represents the nomogram predictions

A Cox proportional hazards model was generated using the most significant parameters identified from the univariate analysis as predictors for ORN (age, V50 Gy volume above 25.4 cm³, and post-radiotherapy dental extraction). An increase in age showed a protective effect (HR = 0.51, 95% CI: 0.27–0.98), while a V50 Gy volume above 25.3 cm³ (HR = 9.87, 95% CI: 2.08–46.73) and post-RT dental extraction (HR = 5.50, 95% CI: 2.24–13.50) was strongly associated with an elevated ORN risk. The model’s predictive performance for 5 years was high, with a C-index of 0.815 and the AUC for 5 years of prediction of ORN was 0.8 (Fig. 4b).

These predictors were incorporated into a nomogram to enable individualized estimation of the 5-year probability of ORN. The nomogram assigns point values to each predictor based on its prognostic importance. These points are then summed to provide a total score, directly correlating with the predicted ORN probability (Fig. 4a).

Further, the Calibration of this model at five years involved bootstrap resampling, yielding a mean absolute error of 0.06 with the 0.9 quantiles of absolute errors at 0.12 (Fig. 4c).

## Discussion

ORN remains one of the most challenging complications following RT for HNC, largely due to its complex management, which often requires surgical intervention [21]. Although the incidence of ORN has decreased with the advent of IMRT in the management of HNC, it still affects approximately 4–8% of patients post-RT [8, 22]. This aligns with the current study result, showing an incidence of 6.7% with a 5-year cumulative incidence of 7.4%.

This study provides insights into predictive factors associated with mandibular ORN after RT. The analysis findings highlight the significance of specific dosimetric parameters and clinical characteristics, which may improve the understanding of ORN risk and aid in developing a predictive model for individualized patient risk assessment.

Firstly, the results emphasize the significance of irradiated mandibular volume, particularly the V50 Gy parameter, as a critical threshold for developing ORN. Patients with V50 Gy volumes exceeding 25.4 cm³ showed a substantially higher incidence of ORN. This finding matches previous studies that identified V50 Gy as one of the most significant variables distinguishing patients who develop mandibular ORN from those who do not, following RT [13, 23]. While increased Dmean was marginally associated with increased risk for ORN, consistent with findings from Gomez et al., neither Dmax nor D2% of the mandible showed a significant association with ORN, as previously assumed [24]. The dose-response relationship with a certain threshold for ORN pathogenesis suggests that larger volumes receiving considerable radiation doses (≥ 50 Gy) may lead to cumulative vascular damage, resulting in hypoxia, fibrosis, and impaired bone remodeling [25]. Interestingly, our emphasis on a dose-volume threshold can be complemented by the recent findings by Rupe et al., who assessed ORN risk by evaluating radiation dose to specific alveolar bone sites [26]. Their prospective study on pre-RT dental extractions observed ORN at mean alveolar doses of approximately 30 Gy, suggesting that clinically meaningful radiation-induced bone injury may still occur even at moderate doses [26]. Importantly, they identified the radiation dose to the extraction socket as a significant predictor of ORN development, highlighting the critical role of localized dosimetry, supporting the notion that even when the overall mandibular dose remains within tolerance limits, a high dose concentrated at vulnerable sites, such as recent extraction locations, can precipitate ORN. These clinical insights are corroborated by preclinical histological studies, which demonstrate that partial radiation doses in the range of 25–30 Gy can induce substantial microvascular damage and inhibit bone remodeling in the mandible [27].

Interestingly, age appeared to have a protective effect, with older patients showing a reduced risk of developing ORN, even after controlling for other variables. In our multivariate model, age still had a small but significant protective effect (HR: 0.96). This observation is in line with previous reports suggesting that ORN predominantly affects younger patients [28, 29]. Although the exact reasons for this phenomenon are unclear, possible explanations may be a follow-up duration bias, younger patients tend to live longer after treatment, giving more time for ORN to manifest. In contrast, older patients may not survive long enough or may die of other causes before ORN becomes clinically evident. Also, the dental status and practices differ by age; younger patients are more likely to have intact dentitions at the time of RT and thus more likely to undergo dental interventions afterward, whereas older patients are often partially/fully edentulous or have already had problematic teeth removed. Biologically, it is also conceivable that age-related differences in bone remodeling and vascularization play a role, for instance, younger bone might mount a more robust but ultimately pathologic remodeling response after radiation, or there may be cohort effects (younger patients today often receive aggressive multimodal therapy).

The mandibular volume in patients with ORN was significantly higher than in those without ORN (67.1 vs. 58.8 cm^3^, *p* = 0.0093). This increase in volume was associated with a marginally significant HR of 1.03 (*p* = 0.058). To our knowledge, this finding has not been previously identified in the literature.

While previous studies did not establish a significant difference in ORN incidence between patients who underwent dental extraction before RT and those who did so afterward [30, 31], our analysis identified post-RT dental extraction as having the most substantial impact on ORN risk, with patients undergoing dental procedures after RT facing a markedly elevated risk. Most patients (10 out of 11) underwent multiple post-RT dental extractions. This finding suggests a possible dose-response-like relationship between the extent of dental trauma and ORN risk, consistent with the hypothesis that repeated disruption of irradiated bone further impairs healing capacity. These findings are in agreement with a growing body of evidence that dental extractions performed after or near the end of the RT course carry a high ORN risk, as emphasized in a recent systematic review, which reported an ORN incidence of about 5.8% in patients who underwent teeth extractions during or after RT (in the current study the incidence was 3.7%) [32]. This is markedly higher than the roughly 2% ORN incidence seen in patients who had teeth removed before starting RT (the incidence in the current study was 1.7%), as previously reported by the same group [17].

The current study supports the recommendations for comprehensive pre-RT dental assessments and interventions, as postponing dental extraction until after RT may significantly increase the incidence of ORN, given the strong association observed, proactive dental management should be an essential component of the care plan for HNSCC patients receiving RT [33, 34].

The predictive model developed in this study, incorporating age, V50 Gy volume, and post-RT dental extraction status, offers a practical tool for estimating the 5-year risk of ORN. The model achieved high predictive accuracy, with a C-index of 0.815 and an area under the curve (AUC) of 0.8, demonstrating strong potential for clinical application. This nomogram enables individualized risk assessment, allowing clinicians to identify high-risk patients who may benefit from additional preventive measures or modified RT protocols. The model’s calibration analysis also validates its reliability, showing good agreement between predicted and observed ORN risks.

It is essential to acknowledge that the dental management strategy at our institution, particularly the timing and indications for pre- versus post-RT extractions, may differ from protocols at other centers, potentially affecting the generalizability of our findings. For example, a more aggressive pre-RT extraction policy might reduce the rate of post-RT dental interventions and thereby modify the observed ORN risk profile. Therefore, when interpreting the impact of dental variables on ORN risk, institutional practices, such as thresholds for prophylactic extractions, timing relative to the start of radiotherapy, and the availability of interdisciplinary oral care, should be considered.

### Study limitation

Despite these promising findings, our study has limitations. Being a retrospective analysis, it is inherently susceptible to selection biases and potential confounding factors. Additionally, our reliance on a single institution dataset may limit the generalizability of these findings. Future multi-institutional studies involving larger cohorts would help validate our predictive model across diverse populations and treatment settings. Although several patient- and treatment-related variables were analyzed, systemic factors such as chronic steroid use or immunosuppression were not assessed. They may represent unmeasured confounders influencing ORN development, as these factors may impair tissue regeneration and bone remodeling, potentially increasing susceptibility to ORN following trauma or dental interventions in irradiated fields. Further, the relative low number of events limited the inclusion of other variables in the multivariable analysis.

In conclusion, this study identifies key dosimetric and clinical predictors of mandibular ORN in HNSCC patients undergoing RT. The V50 Gy volume threshold, patient age, and post-RT dental extractions emerged as significant risk factors and were incorporated into a predictive nomogram to facilitate risk stratification and personalized treatment planning. Our findings emphasize the need for careful RT planning and proactive dental management to mitigate ORN risk and ultimately improve quality of life in patients receiving RT for head and neck cancers.

## Electronic supplementary material

Below is the link to the electronic supplementary material.

Supplementary Material 1

## Data Availability

No datasets were generated or analysed during the current study.
