# Supplementary material for: Predictive modelling of mandibular osteoradionecrosis in head and neck cancer patients: clinical and dosimetric insights
Source: Clin Oral Investig. 2025 May 26;29(6):313. doi: 10.1007/s00784-025-06385-3 (PMC12106553; doi:10.1007/s00784-025-06385-3)
Supplement: Supplementary file 1 — Supplementary Material 1 [file 784_2025_6385_MOESM1_ESM.docx]

**Appendix 1**

STROBE Checklist of items that should be included in reports of ***cohort studies***

|  | Item No | Recommendation | Page No |
| --- | --- | --- | --- |
| **Title and abstract** | 1 | (*a*) Indicate the study’s design with a commonly used term in the title or the abstract | 1 |
|  |  | (*b*) Provide in the abstract an informative and balanced summary of what was done and what was found | 2-3 |
| Introduction | | | |
| Background/rationale | 2 | Explain the scientific background and rationale for the investigation being reported | 4-5 |
| Objectives | 3 | State specific objectives, including any prespecified hypotheses | 5 |
| Methods | | | |
| Study design | 4 | Present key elements of study design early in the paper | 6 |
| Setting | 5 | Describe the setting, locations, and relevant dates, including periods of recruitment, exposure, follow-up, and data collection | 6-10 |
| Participants | 6 | (*a*) Give the eligibility criteria, and the sources and methods of selection of participants. Describe methods of follow-up | 6 |
|  |  | (*b*) For matched studies, give matching criteria and number of exposed and unexposed | NA |
| Variables | 7 | Clearly define all outcomes, exposures, predictors, potential confounders, and effect modifiers. Give diagnostic criteria, if applicable | 8-9 |
| Data sources/ measurement | 8* | For each variable of interest, give sources of data and details of methods of assessment (measurement). Describe comparability of assessment methods if there is more than one group | 7-8 |
| Bias | 9 | Describe any efforts to address potential sources of bias | 9 |
| Study size | 10 | Explain how the study size was arrived at | NA |
| Quantitative variables | 11 | Explain how quantitative variables were handled in the analyses. If applicable, describe which groupings were chosen and why | 9 |
| Statistical methods | 12 | (*a*) Describe all statistical methods, including those used to control for confounding | 9 |
|  |  | (*b*) Describe any methods used to examine subgroups and interactions | NA |
|  |  | (*c*) Explain how missing data were addressed | NA |
|  |  | (*d*) If applicable, explain how loss to follow-up was addressed | NA |
|  |  | (*e*) Describe any sensitivity analyses | 9 |
| Results | | |  |
| Participants | 13* | (a) Report numbers of individuals at each stage of study—eg numbers potentially eligible, examined for eligibility, confirmed eligible, included in the study, completing follow-up, and analysed | 10 |
|  |  | (b) Give reasons for non-participation at each stage | 10 |
|  |  | (c) Consider use of a flow diagram | 10 |
| Descriptive data | 14* | (a) Give characteristics of study participants (eg demographic, clinical, social) and information on exposures and potential confounders | 11 |
|  |  | (b) Indicate number of participants with missing data for each variable of interest | 10-11 |
|  |  | (c) Summarise follow-up time (eg, average and total amount) | 10-11 |
| Outcome data | 15* | Report numbers of outcome events or summary measures over time | 10 |

| Main results | 16 | (*a*) Give unadjusted estimates and, if applicable, confounder-adjusted estimates and their precision (eg, 95% confidence interval). Make clear which confounders were adjusted for and why they were included | 13-14 |
| --- | --- | --- | --- |
|  |  | (*b*) Report category boundaries when continuous variables were categorized | 15 |
|  |  | (*c*) If relevant, consider translating estimates of relative risk into absolute risk for a meaningful time period | NA |
| Other analyses | 17 | Report other analyses done—eg analyses of subgroups and interactions, and sensitivity analyses | 15-16 |
| Discussion | | | |
| Key results | 18 | Summarise key results with reference to study objectives | 16-21 |
| Limitations | 19 | Discuss limitations of the study, taking into account sources of potential bias or imprecision. Discuss both direction and magnitude of any potential bias | 21 |
| Interpretation | 20 | Give a cautious overall interpretation of results considering objectives, limitations, multiplicity of analyses, results from similar studies, and other relevant evidence | 20-21 |
| Generalisability | 21 | Discuss the generalisability (external validity) of the study results | 20-21 |
| Other information | | | |
| Funding | 22 | Give the source of funding and the role of the funders for the present study and, if applicable, for the original study on which the present article is based | NA |

### **Appendix 2**

TRIPOD checklist

| **Section/Topic** | **Item** | **Checklist Item** | **Page** |
| --- | --- | --- | --- |
| **Title and abstract** | | | |
| Title | 1 | Identify the study as developing and/or validating a multivariable prediction model, the target population, and the outcome to be predicted. | **1** |
| Abstract | 2 | Provide a summary of objectives, study design, setting, participants, sample size, predictors, outcome, statistical analysis, results, and conclusions. | 2 |
| **Introduction** | | | |
| Background and objectives | 3a | Explain the medical context (including whether diagnostic or prognostic) and rationale for developing or validating the multivariable prediction model, including references to existing models. | 4-5 |
|  | 3b | Specify the objectives, including whether the study describes the development or validation of the model or both. | 5 |
| **Methods** | | | |
| Source of data | 4a | Describe the study design or source of data (e.g., randomized trial, cohort, or registry data), separately for the development and validation data sets, if applicable. | 6 |
|  | 4b | Specify the key study dates, including start of accrual; end of accrual; and, if applicable, end of follow-up. | 6 |
| Participants | 5a | Specify key elements of the study setting (e.g., primary care, secondary care, general population) including number and location of centres. | 6 |
|  | 5b | Describe eligibility criteria for participants. | 6 |
|  | 5c | Give details of treatments received, if relevant. | 6 |
| Outcome | 6a | Clearly define the outcome that is predicted by the prediction model, including how and when assessed. | 8 |
|  | 6b | Report any actions to blind assessment of the outcome to be predicted. | NA |
| Predictors | 7a | Clearly define all predictors used in developing or validating the multivariable prediction model, including how and when they were measured. | 6 |
|  | 7b | Report any actions to blind assessment of predictors for the outcome and other predictors. | NA |
| Sample size | 8 | Explain how the study size was arrived at. | NA |
| Missing data | 9 | Describe how missing data were handled (e.g., complete-case analysis, single imputation, multiple imputation) with details of any imputation method. | NA |
| Statistical analysis methods | 10a | Describe how predictors were handled in the analyses. | 8-9 |
|  | 10b | Specify type of model, all model-building procedures (including any predictor selection), and method for internal validation. | 8-9 |
|  | 10d | Specify all measures used to assess model performance and, if relevant, to compare multiple models. | 8 |
| Risk groups | 11 | Provide details on how risk groups were created, if done. | NA |
| **Results** | | | |
| Participants | 13a | Describe the flow of participants through the study, including the number of participants with and without the outcome and, if applicable, a summary of the follow-up time. A diagram may be helpful. | 9 |
|  | 13b | Describe the characteristics of the participants (basic demographics, clinical features, available predictors), including the number of participants with missing data for predictors and outcome. | 9 |
| Model development | 14a | Specify the number of participants and outcome events in each analysis. | 9 |
|  | 14b | If done, report the unadjusted association between each candidate predictor and outcome. | 12 |
| Model specification | 15a | Present the full prediction model to allow predictions for individuals (i.e., all regression coefficients, and model intercept or baseline survival at a given time point). | 11-14 |
|  | 15b | Explain how to the use the prediction model. | 15 |
| Model performance | 16 | Report performance measures (with CIs) for the prediction model. | 15 |
| **Discussion** | | | |
| Limitations | 18 | Discuss any limitations of the study (such as nonrepresentative sample, few events per predictor, missing data). | 18 |
| Interpretation | 19b | Give an overall interpretation of the results, considering objectives, limitations, and results from similar studies, and other relevant evidence. | 16-18 |
| Implications | 20 | Discuss the potential clinical use of the model and implications for future research. | 18-19 |
| **Other information** | | | |
| Supplementary information | 21 | Provide information about the availability of supplementary resources, such as study protocol, Web calculator, and data sets. | NA |
| Funding | 22 | Give the source of funding and the role of the funders for the present study. | NA |

### **Appendix 3**

Detailed characteristics for Patients with ORN

| Patient | Age | Sex | Tumor Location | Stage | Therapy | Time of ORN (months) | Grade |
| --- | --- | --- | --- | --- | --- | --- | --- |
| 1 | 75 | M | Salivary gland | pT4a pN2b | Adjuvant | 11 | G3 |
| 2 | 63 | M | Oropharynx | cT4 cN0 | Primary | 50 | G2 |
| 3 | 47 | M | Nasopharynx | cT1 cN2c | Primary | 3 | G2 |
| 4 | 67 | M | CUP | pTx pN2b | adjuvant | 30 | G1 |
| 5 | 66 | M | Oral cavity | pT4a pN1 | adjuvant | 60 | G3 |
| 6 | 58 | M | Oral cavity | pT3 pN0 | adjuvant | 64 | G2 |
| 7 | 54 | M | Oral cavity | pT2 pN1 | adjuvant | 4 | G3 |
| 8 | 61 | F | Oropharynx | pT2 pN2b | adjuvant | 92 | G2 |
| 9 | 69 | F | Oral cavity | pT3 pN0 | adjuvant | 30 | G2 |
| 10 | 66 | M | Hypopharynx | pT3 pN1 | adjuvant | 149 | G2 |
| 11 | 63 | F | Oral cavity | pT3 pN2c | Adjuvant | 30 | G2 |
| 12 | 69 | F | Oral cavity | pT2 pN1 | Adjuvant | 34 | G2 |
| 13 | 62 | M | Salivary Gland | pT1 pN2b | adjuvant | 6 | G1 |
| 14 | 82 | F | Salivary Gland | pT1 pN2a | adjuvant | 64 | G2 |
| 15 | 66 | M | Oropharynx | pT2 pN2 | adjuvant | 15 | G2 |
| 16 | 74 | F | Oral cavity | pT1 pN2b | adjuvant | 19 | G2 |
| 17 | 71 | M | Oropharynx | pT2 pN0 | adjuvant | 65 | G1 |
| 18 | 70 | M | Oropharynx | T3-4 N+ M0 | primary | 18 | G2 |
| 19 | 61 | M | Oropharynx | pT3 pN1 | adjuvant | 11 | G2 |
| 20 | 57 | F | Oropharynx | cT3 N1 | primary | 6 | G1 |
